# A267 IDENTIFYING ENTEROBACTERIACEAE VIRULENCE GENES ASSOCIATED WITH ACTIVE DISEASE IN ULCERATIVE COLITIS PATIENTS USING CULTURE-DEPENDENT AND -INDEPENDENT APPROACHES

**DOI:** 10.1093/jcag/gwac036.267

**Published:** 2023-03-07

**Authors:** D Tertigas, F Rinawi, A Griffiths, M Surette

**Affiliations:** 1 Department of Biochemistry and Biomedical Sciences, McMaster University, Hamilton, Canada; 2 The Ruth and Bruce Rappaport Faculty of Medicine, Technion - Israel Institute of Technology, Haifa, Israel; 3 SickKids Inflammatory Bowel Disease Center, The Hospital for Sick Children, University of Toronto, Toronto; 4 Department of Medicine, McMaster University, Hamilton, Canada

## Abstract

**Background:**

The prevalence of inflammatory bowel disease (IBD) in Canada is among the highest in the world and is estimated to affect 1 in 100 Canadians by 2030. Ulcerative colitis (UC) is a type of IBD characterized by mucosal inflammation of the large intestine. UC is believed to arise through a complex interplay of the host immune responses and changes in the gut microbiota in a genetically susceptible individual. Therapies targeting the gut microbiota, such as antibiotics and fecal microbiota transplantation (FMT), have been effective in treating UC, suggesting infectious triggers should be explored.

**Purpose:**

Some data suggests the development of UC can be driven by pathogenic bacteria of the family *Enterobacteriaceae*, which can carry virulence genes important for colonizing the gut (e.g. *fimH*) and disrupting the intestinal epithelium (e.g. *hylA*). However, many studies have focused on a single species (e.g. *Escherichia coli*) and thereby underestimate the importance of these virulence genes that are shared across the *Enterobacteriaceae* family. **I aim to investigate whether specific virulence genes contribute to disease activity in some patients with UC and to show that these virulence genes are carried by strains of many *Enterobacteriaceae* species.**

**Method:**

UC patient stool samples were collected throughout enrolment in randomized control trials of FMT for adult UC and microbiome studies in early-onset pediatric UC. The stool samples were cultured on MacConkey agar to enrich for *Enterobacteriaceae.* Samples from before and after treatment were sent for targetted cultured-enriched metagenomic sequencing and strains were isolated from baseline samples only for whole genome sequencing. The taxonomy of each genome and taxonomic composition of each metagenome were annotated along with virulence genes and antimicrobial resistance genes. Phenotypic assays of cultured isolates were used to capture diversity and virulence activity.

**Result(s):**

Approximately 7500 colonies from UC patient stool samples were isolated and phenotyped. Based on the initial screens, 130 isolates were selected to comprise our *Enterobacteriaceae* strain collection. Across all patient samples, we detected 19 different species of the *Enterobacteriaceae* family across six genera from the genomic and metagenomic data. We identified virulence genes found across multiple species from the *Enterobacteriaceae* family within genomes and metagenomes, and by performing phenotypic assays of the cultured isolates.

**Conclusion(s):**

Further exploration of the distribution of these virulence genes in UC patients during active disease or remission and healthy controls can provide insight into the pathogenesis of UC. Identifying infectious agents in even a subset of UC patients will allow for more targeted diagnosis and treatment approaches.

**Disclosure of Interest:**

None Declared

